# Supplementary material for: Assessment of Binge Eating Behavior, Body Shape Concerns, and Associated Factors among Female Adolescents of Northern Saudi Arabia: A Cross-Sectional Study
Source: Nutrients. 2024 Sep 13;16(18):3082. doi: 10.3390/nu16183082 (PMC11435104; doi:10.3390/nu16183082)
Supplement: Supplementary file 1 [file nutrients-16-03082-s001.zip › Supplementary Table S1.pdf]

Supplementary Table S1. Frequency and proportion of participants' responses in binge eating scale (*n* = 400)

Variable	0	1	2	3
	n (%)	n (%)	n (%)	n (%)
BES (Q1): Awareness of my appearance and body weight	217 (54.2)	115 (28.8)	30 (7.5)	38 (9.5)
BES (Q2): Eating much more rapidly than normal during binge episodes.	300 (75.0)	51 (12.7)	41 (10.3)	8 (2.0)
BES (Q3): Loss of control overeating (Impulse to eat)	313 (78.2)	39 (9.8)	30 (7.5)	18 (4.5)
BES (Q4): Habit of eating when I'm bored	211 (52.7)	121 (30.3)	46 (11.5)	22 (5.5)
BES (Q5): Eating only when I am hungry	236 (59.0)	75 (18.8)	53 (13.3)	36 (9.0)
BES (Q6): Feelings of shame or guilt regarding eating habits*	296 (74.0)	69 (17.3)	35 (8.7)	N/A
BES (Q7): I maintain control over my eating habits while dieting, even after episodes of overeating.	302 (75.5)	55 (13.7)	25 (8.8)	8 (2.0)
BES (Q8): Marked distress about binge eating episodes.	235 (58.8)	28 (7.0)	98 (24.5)	39 (9.8)
BES (Q9): Intake of high or low calories on regular basis	245 (61.2)	83 (20.8)	65 (16.3)	7 (1.8)
BES (Q10): I can typically stop eating when I choose to	341 (85.3)	27 (6.8)	23 (5.8)	9 (2.3)
BES (Q11): I have no problem stopping eating when I feel full.	340 (85.0)	34 (8.5)	22 (5.5)	4 (1.0))
BES (Q12): I seem to eat when I am with others (family and social gatherings) the same way when I am alone.	281 (70.3)	66 (16.5)	30 (7.5)	23 (5.8)
BES (Q13): I eat three meals a day with a snack sometimes.	251 (62.8)	63 (15.8)	54 (13.4)	32 (8.0)
BES (Q14): Experiencing significant distress or impairment in daily functioning due to binge eating episodes	297 (74.3)	36 (9.0)	32 (8.0)	35 (8.7)
BES (Q15): Hiding food to eat later in secret	294 (73.5)	47 (11.8)	51 (12.8)	8 (2.0)
BES (Q16): Consuming large amounts of food even in the absence of physical hunger*.	289 (72.2)	63 (15.8)	48 (12.0)	N/A

0 – No problem, 1 – Mild, 2 – Medium, 3 – High; * Question 6 and 16 included only 3 responses according to the BES scale.
