# Supplementary material for: Assessment of Binge Eating Behavior, Body Shape Concerns, and Associated Factors among Female Adolescents of Northern Saudi Arabia: A Cross-Sectional Study
Source: Nutrients. 2024 Sep 13;16(18):3082. doi: 10.3390/nu16183082 (PMC11435104; doi:10.3390/nu16183082)
Supplement: Supplementary file 1 [file nutrients-16-03082-s001.zip › Supplementary Table S2.pdf]

Supplementary Table S2. Participants responses in body shape questionnaire-short version (BSQ-8C) (*n* = 400)

Parameter	Not at all n (%)	Rarely n (%)	Sometimes n (%)	Often n (%)	Usually n (%)	All the time n (%)
Are you afraid of becoming fat (or fatter)?	180 (45.0)	59 (14.8)	80 (20.0)	24 (6.0)	25 (6.3)	32 (8.0)
Did feeling full make you feel obese (e.g., after eating a large meal)?	171 (42.8)	92 (23.0)	58 (14.5)	16 (4.0)	14 (3.5)	14 (12.3)
Were you hindered in your ability to concentrate or focus due to thoughts about your appearance (e.g., while watching TV)	253 (63.2)	65 (16.2)	41 (10.3)	4 (1.0)	11 (2.8)	26 (6.5)
Have you thought about gastrectomy or removal of some fat from your body?	307 (76.7)	20 (5.0)	42 (10.5)	14 (3.5)	13 (3.3)	4 (1.0)
Experienced feelings of being too large or overweight	305 (76.2)	23 (5.8)	20 (5.0)	28 (7.0)	10 (2.5)	14 (3.5)
Perceived appearance as indicative of a lack of self-control	290 (72.4)	17 (4.3)	24 (6.0)	23 (5.8)	25 (6.3)	21 (5.2)
Feeling negatively about appearance upon seeing reflection (mirror or shop windows	339 (84.7)	11 (2.8)	14 (3.5)	12 (3.0)	6 (1.5)	18 (4.5)
Have you had a special self-awareness about how you look when you're in the company of others?	169 (42.2)	30 (7.5)	34 (8.5)	49 (12.3)	16 (4.0)	102 (25.5)
